# Population-Based Detection of Cancer Cases Using Digital Platforms in Mangalagiri Mandal of Guntur District, Andhra Pradesh

**DOI:** 10.7759/cureus.34785

**Published:** 2023-02-08

**Authors:** Dhrubajyoti J Debnath, Arti Gupta, Navya Krishna Naidu, Vinoth Kumar Kalidoss, Rakesh Kakkar

**Affiliations:** 1 Department of Community and Family Medicine, All India Institute of Medical Sciences, Mangalagiri, IND; 2 Department of Community and Family Medicine, All India Institute of Medical Sciences, Bathinda, IND

**Keywords:** population, prevalence, incidence, cancer registry, cancer

## Abstract

Background and objectives

Cancer is one of the major causes of illness and mortality in India. The cancer burden in India will roughly triple over the next 20 years. Population-based cancer registries (PBCRs) are crucial not only for formulating cancer control policies and assessing their effectiveness but also offer essential inputs for programs aimed at preventing cancer. The state of Andhra Pradesh after the division in 2014 has been left with no PBCR. Hence, with the aim to pilot the development of a population-based cohort of cancer cases with digital reporting and monitoring mechanisms, this study was carried out with the objective of finding the prevalence and incidence of various types of cancer in Mangalagiri Mandal, Guntur district, Andhra Pradesh.

Materials and methods

A cross-sectional survey during the period January 2021 to June 2022 was conducted to actively search for cancer cases among 160303 people residing in 42639 households in the Mangalagiri Mandal, Guntur district, State of Andhra Pradesh, India. The respondents were asked if anyone in the household had cancer and the location was mapped, the information was recorded by the Accredited Social Health Activist (ASHA) workers in the pre-tested questionnaire. The total number of cancer cases identified was 107.

Results

Mangalagiri Mandal had 24 cases of cancer among men and 83 cases of cancer among women as recorded during the study period. Most of the male (37.5%) and female (31.3%) cancer cases were in Stage 2 of cancer and the majority of them (91.6%, 94%) had completed their treatment and were on regular follow-up. The most common cancer among both sexes was breast cancer (5.45 per million population). Oral cancer was the most prevalent in men (0.747 per million of the population) whereas, breast cancer (4.253 per million population) was the most prevalent among females. A total of 47.1% of the males had cancer at sites that were related to tobacco usage.

Conclusion

The data on cancer incidence and prevalence along with the socio-demographic profile is essential to know the burden of cancer. This baseline data can be used in planning cancer control activities and knowing the future trend of cancer in the Mangalagiri Mandal, in the new state of Andhra Pradesh.

## Introduction

One of the major causes of illness and death in India is cancer. Over one million of 14 million newly diagnosed cases of cancer worldwide came from India. Population-based cancer registries (PBCRs) are essential for developing cancer control strategies and evaluating their effectiveness. PBCRs offer essential inputs for programs aimed at preventing cancer. In India, the burden of cancer varies within the regions which poses great challenges in its prevention and control. PBCRs must be constructed uniformly across the nation while taking into account the regional variety and population distribution. The integration of medical information from hospitals, cancer treatment facilities, and other healthcare facilities with PBCRs is quite helpful [1,2].

Global Cancer Observatory (GLOBOCAN) predicts that India's cancer burden will roughly treble in the next 20 years, according to International Agency for Research on Cancer (IARC) forecast [3]. Worldwide, the incidence and mortality of cancer are quickly increasing, especially in low- and middle-income nations. It is imperative that nations establish and put into practice cancer control measures. However, just one in five low- and middle-income nations have the data required to inform policy [4].

Digitalized capturing of complete population-based data on cancer cases in a defined geographical area especially in rural areas is a challenging as well as a novel approach. Hospital-based data is not a reliable estimate of area-specific burden. The population-based true burden of cancer, with organs involved, likely etiology for rising burden, health behavior, and related socioeconomic details for cancer cases will be estimated. Early detection of cancer cases can improve the survival time of these cases. Further, follow-up of each and every cancer case will generate data on survival outcomes at a population level [5].

As per the Indian Council of Medical Research (ICMR) National Centre for Disease Informatics and Research (NCDIR) 2020 report, the state of Andhra Pradesh after the division in 2014 has been left with no population-based or hospital-based cancer registries (HBCRs). The HBCR is at Rural Development Trust, Bathalapalle, Andhra Pradesh [6]. The Nellore district of Andhra Pradesh had a PBCR which is projecting 20 years old data [7].

A little over 58,000 cancer cases were reported in Andhra Pradesh, according to the ICMR. The research also reveals that 29,244 people lost their lives to cancer in 2016. Cervical and breast cancers, which are easily identified and prevented, are prevalent among women in the state and the numbers are rising. Due to the exorbitant expense of cancer treatment at private institutions, the average person is giving up treatment for the disease. The establishment of hospitals and the provision of free healthcare are crucial. The availability of affordable treatment will increase the number of diagnoses and enable us to save more lives [8]. It is vital for the state of Andhra Pradesh to generate population-based cancer information for policymakers, academicians, and researchers toward prevention and cure.

Hence, to have population-based cancer data, the study aimed to pilot the development of a population-based cohort of cancer cases with a digital reporting and monitoring mechanism for the Mangalagiri, Guntur District, State of Andhra Pradesh, India. Information collected was related to the diagnosis of cancer, treatment, follow-up, survival, and any other important issue of cancer patients. This led to the identification of the cancer burden, the needs of cancer patients, reasons for dropouts of treatment, and the need for palliative care.

With this background, the study was carried out with the objective of finding the prevalence and incidence of various types of cancer in Mangalagiri Mandal, Guntur district, Andhra Pradesh.

## Materials and methods

Study design and population

Our sample population consisted of people residing in the Mangalagiri Mandal, Guntur district, State of Andhra Pradesh, India. Cancer is not a notifiable disease in India. A cross-sectional survey was conducted to actively search for cancer cases. There were approximately 42639 households in the Mangalagiri Mandal [9]. The respondents were asked whether any of the family members were presently suffering from cancer. If anyone in the household had cancer, the information was recorded in the pre-tested questionnaire. The data were based on responses to this survey. The survey was conducted during the period from January 2021 to June 2022.

Sample size

Households residing in the Mangalagiri Mandal, Guntur district, State of Andhra Pradesh, India were covered in the survey with a population of 160303 to identify the diagnosed cases of cancer in support of Accredited Social Health Activists (ASHAs). The total number of cancer cases identified was 107.

Study tools and data collection

A pre-tested questionnaire was used as the study instrument. The sociodemographic variables, information regarding the site of cancer, staging of cancer, and treatment taken were covered. The latitude and longitude of the homes of cancer cases were taken for the purpose of mapping these cases. The information on cancer cases was collected by involving grass root level community healthcare workers and ASHA workers.

Confidentiality of personal data was ensured strictly throughout the study.

Tobacco use is a cause of several cancers. The cancer sites with sufficient evidence of carcinogenicity related to tobacco exposure in humans are the cancer of the lung, oral cavity, pharynx, larynx, pancreas, urinary bladder, renal pelvis and ureter, nasal cavity and nasal sinuses, esophagus, stomach, liver, kidney, uterine cervix, and bone marrow (myeloid leukemia) [10]. The data of these cancers were analyzed to determine the tobacco-related exposure associated with cancer.

Ethical considerations

This study was approved by the Institute Ethics Committee (IEC) of All India Institute of Medical Sciences, Mangalagiri, Andhra Pradesh vide IEC certificate AIIMS/MG/IEC/2020-21/23 dated: 01/09/2020. Informed consent was taken from each participant prior to collecting data after explaining the purpose of the study and assuring them that their responses would be kept confidential.

Data analysis

The data were systematically entered into an MS Excel database. Continuous variables were expressed as mean ± standard deviation, and median (minimum-maximum). The nominal data were expressed as numbers and percentages. The primary outcome of interest was the prevalence and incidence of the different types of cancer in Mangalagiri Mandal in males and females.

## Results

Table 1 shows that Mangalagiri Mandal had 24 cases of cancer among men and 83 cases of cancer among women as recorded from July 2021 to June 2022. In males, the age of cancer cases ranged from 32 to 78 years with a mean (SD) of 55.1 (13.8) years. In females, the age of cancer cases ranged from 22 to 81 years with a mean (SD) of 55.2 (11.7) years. The median age among males was 58.5 years and for females was 54 years.

The majority (8, 33.3%) of male cases were in the 60-69 years age group, followed by the 30-39 age group. The majority of female cases (24, 28.9%) were in the 50-59 age group, followed by the 40-49 years (23, 27.7%) age group.

Most of the male cancer patients were in Stage 2 of cancer (9, 37.5%) followed by Stage 1 (7, 29.2%). The majority of the female patients were in Stage 2 of cancer (26, 31.3%) followed by Stage 4 (22, 26.5%). The majority of cancer cases in men (22, 91.6%) had received their complete therapy for cancer and were on regular follow-ups (22, 91.7%). Among the women who had cancer, almost all (78, 94%) had completed their treatment for cancer, and 80 of them i.e., 96.4% had undergone regular check-ups. Two (8.3%) males and four (4.8%) females could not take regular follow up due to financial constraints.

**Table 1:** Characteristics of the cancer cases in Mangalagiri Mandal, Guntur district, Andhra Pradesh AYUSH: Ayurveda, Yoga and Naturopathy, Unani, Siddha, and Homeopathy; IQR: interquartile range; SD: standard deviation

Variable	Characteristics	Male (n=24)	Female (n=83)
Age, years	Range (in years)	32.0-78.0	22.0-81.0
	Mean age (SD) (in years)	55.1 (13.8)	55.2 (11.7)
	Median age (IQR), in years	58.5 (44.0-65.5)	54.0 (48.0-65.0)
Age group, (years)	< 30 (N, %)	0 (0)	1 (1.2%)
	30-39	5 (20.8%)	5 (6%)
	40-49	3 (12.5%)	23 (27.7%)
	50-59	4 (16.7%)	24 (28.9)
	60-69	8 (33.3%)	18 (21.7%)
	> 70 (N, %)	4 (16.7%)	12 (14.5%)
Cancer stage	1 (N, %)	7 (29.2)	20 (24.1)
	2	9 (37.5)	26 (31.3)
	3	3 (12.5)	15 (18.1)
	4 (N, %)	5 (20.8)	22 (26.5)
Treatment for cancer as recommended	Completed (N, %)	22 (91.6)	78 (94)
	Partial treatment taken	1 (4.2)	4 (1.8)
	Did not take any treatment (N, %)	1 (4.2)	1 (1.2)
Regular follow-up after cancer was diagnosed	Yes (N, %)	22 (91.7)	80 (95.2)
	No (N, %)	2 (8.3)	4 (4.8)
Treatment for cancer taken from AYUSH practitioners	Yes (N, %)	0 (0)	3 (2.8)
	No (N, %)	24 (100)	80 (97.2)

**Figure 1:**
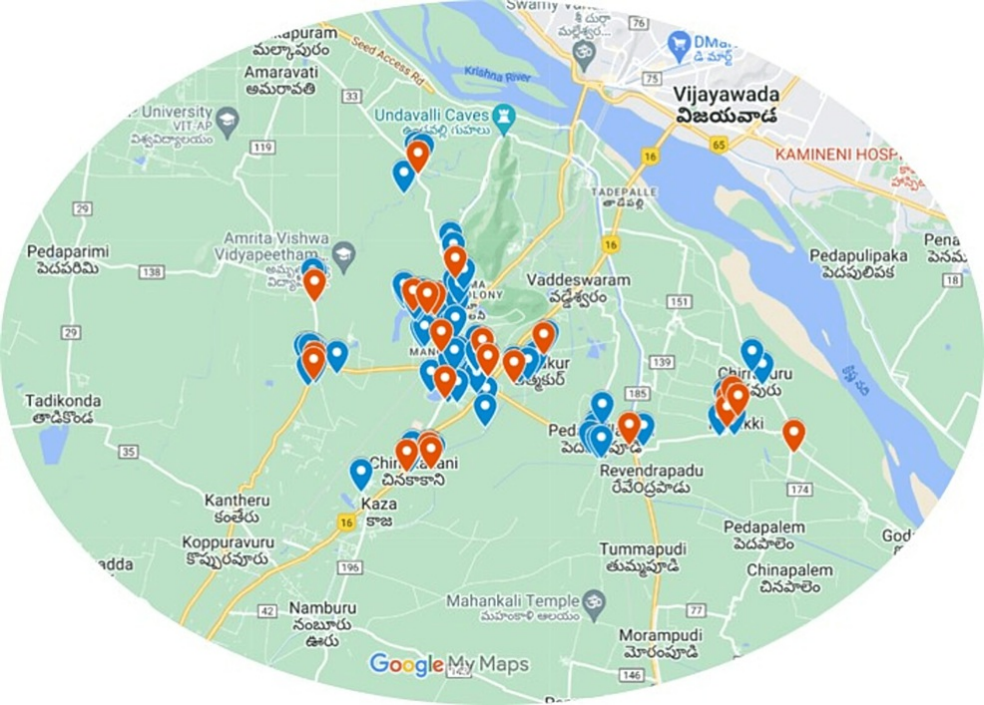
Cancer cases mapping on the map of Mangalagiri Mandal, Guntur, Andhra Pradesh Blue pointers Female, Red pointers Male

Figure 1 shows the geolocation of mapped cancer patients in Mangalagiri Mandal. Twenty-four cases of cancer among men and 83 cases of cancer among women were identified.

The most common cancer among both sexes during the study period in Mangalagiri mandal was breast cancer (5.45 per million population) with 4.253 per million population among females. This was followed by cervical cancer (1.924 per million population), ovarian cancer (1.443 per million population), oral cancer (1.282 per million population), and tongue cancer (0.962 per million population). The overall prevalence rate of cancer in all sites among both sexes is 17.152 per million population. The incidence of breast cancer and oral cancer was 0.802 per million population, in 2021, making them the top cancers most commonly occurring among both sexes. The incidence of cancer at all sites in the year 2021 was 3.206 per million population (Table 2).

**Table 2:** Prevalence and incidence of cancer cases in both sexes residing in Mangalagiri Mandal of Guntur district, Andhra Pradesh CML: chronic myelogenous leukemia

Type of cancer	Prevalence of cancer cases in Mangalagiri Mandal during the period July 2021 to June 2022 (per million population)	Incidence of cancer (2021) Mangalagiri (per million population)
All sites	17.152	3.206
Breast cancer	5.45	0.802
Cervical cancer	1.924	0
Ovarian cancer	1.443	0.16
Oral cancer	1.282	0.802
Tongue cancer	0.962	0.16
Lung cancer	0.641	0
Vaginal cancer	0.481	0.16
Oesophagus cancer	0.481	0.16
Brain cancer	0.481	0.16
Larynx cancer	0.321	0
Post cricoid cancer	0.321	0.16
CML	0.321	0
Acute lymphoblastic leukemia (ALL)	0.16	0
Anus cancer	0.16	0
Uterine cancer	0.16	0
Bladder cancer	0.16	0
Caecum cancer	0.16	0.16
Carcinoid tumor	0.16	0
Colon cancer	0.16	0.16
Lip cancer	0.16	0
Lymphoma cancer	0.16	0
Multiple myeloma	0.16	0
Pancreas cancer	0.16	0
Pharynx cancer	0.16	0
Rectal cancer	0.16	0
Skin cancer	0.16	0
Small intestine cancer	0.16	0
Soft tissue sarcoma	0.16	0
Stomach cancer	0.16	0.16
Thyroid cancer	0.16	0.16
Unknown primary with neck secondaries	0.16	0

In Mangalagiri Mandal, oral cancer was the most prevalent cancer in men during the period July 2021 to June 2022 (0.747 per million of the population), followed by tongue cancer (0.498 per million), lung cancer (0.249 per million), and chronic myeloid leukemia (0.249 per million population). The overall prevalence rate of cancer at all sites among males was 2.986 per million population. Oral cancer had the highest incidence rate of 0.622 per million population in 2021, followed by tongue, brain, and caecum cancer, which all had similar incidence rates of 0.124 per million population. In Mangalagiri Mandal, the incidence of cancer at all sites among men in the year 2021 was 0.995 per million people (Table 3).

**Table 3:** Prevalence and incidence of cancer cases in males residing in Mangalagiri Mandal of Guntur district, Andhra Pradesh

Type of cancer	Prevalence of cancer cases in males during the period July 2021 to June 2022 (per million population)	Incidence of cancer cases in females in the year 2021 (per million population)
All sites	2.986	0.995
Oral cancer	0.747	0.622
Tongue cancer	0.498	0.124
Lung cancer	0.249	0
Chronic myeloid leukemia	0.249	0
Brain cancer	0.124	0.124
Caecum cancer	0.124	0.124
Carcinoid tumor	0.124	0
Larynx cancer	0.124	0
Lip cancer	0.124	0
Multiple myeloma	0.124	0
Oesophagus cancer	0.124	0
Skin cancer	0.124	0
Small intestine cancer	0.124	0
Unknown primary with neck secondaries	0.124	0

The most prevalent cancer among females in Mangalagiri Mandal was breast cancer (4.253 per million population), which was followed by cervical cancer (1.501 per million population), ovarian cancer (1.126 per million population), and vaginal cancer (0.375 per million population). The overall prevalence rate of cancer at all sites among women was 10.383 per million population. Breast cancer had the highest incidence rate of 0.625 per million population in 2021, followed by ovarian, vaginal, thyroid, and other cancers as shown in Table 4. The total incidence of cancer among females in the year 2021 was 1.501 per million population.

**Table 4:** Prevalence and incidence of cancer cases in females residing in Mangalagiri Mandal of Guntur district, Andhra Pradesh

Type of cancer	Prevalence of cancer cases in females during the period July 2021 to June 2022 (per million population)	Incidence of cancer cases in the year 2021 (per million population)
All sites	10.383	1.501
Breast cancer	4.253	0.625
Cervical cancer	1.501	0
Ovarian cancer	1.126	0.125
Vaginal cancer	0.375	0.125
Oral cancer	0.25	0
Lung cancer	0.25	0
Tongue cancer	0.25	0
Brain cancer	0.25	0
Oesophagus cancer	0.25	0.125
Post cricoid cancer	0.25	0.125
Acute lymphoblastic leukemia	0.125	0
Anal cancer	0.125	0
Bladder cancer	0.125	0
Colon cancer	0.125	0.125
Larynx cancer	0.125	0
Lymphoma cancer	0.125	0
Pancreas cancer	0.125	0
Pharynx cancer	0.125	0
Rectal cancer	0.125	0
Soft tissue sarcoma	0.125	0
Stomach cancer	0.125	0.125
Thyroid cancer	0.125	0.125
Uterine cancer	0.125	0

Table 5 shows that 47.1% of the males had cancer at sites that were related to tobacco usage whereas there was no cancer detected in females with evidence related to tobacco usage.

**Table 5:** Tobacco use in cases who had cancer at sites with sufficient evidence of carcinogenicity related to tobacco exposure

Variable	Characteristics	Male (n=17)	Female (n=29)
Tobacco use	Yes (N, %)	8 (47.1)	0 (0)
	No (N, %)	9 (52.9)	29 (100)

## Discussion

Geo-mapping and creating a PBCR for Mangalagiri Mandal for Andhra Pradesh is the first attempt towards a PBCR for Andhra Pradesh. PBCRs are important resources for tracking the incidence and prevalence of cancer in a specific population. Missing PBCRs can result in a lack of accurate and detailed information about cancer rates in a certain area, making it difficult to understand the burden of the disease and to plan and evaluate cancer control efforts. This can also affect the ability to monitor trends over time and identify disparities in cancer rates among different subpopulations. Also, missing PBCRs can also affect the ability to identify areas with higher cancer rates, which can be important for allocating resources and targeting cancer control efforts. And also it can affect the ability to conduct cancer research and identify potential risk factors for cancer in a specific population.

During the period of one year from July 2021 to June 2022, the prevalence of all sites cancer cases per million population was 1.715 among both sexes, 2.986 among males, and 10.383 among females. Among both males and females, breast cancer had the highest prevalence of 5.45 per million population followed by cervical cancer (1.924/1000000), ovarian cancer (1.43/1000000 population), oral cancer (1.282/1000000), and tongue cancer (0.962/1000000) population. The prevalence and incidence of cancer were higher among females as compared to males. Majority of the cancer cases occurred in the 50-59 years age - group followed by the 40-49 and 60-69 age groups.

In the present study, we found that the leading sites of cancer in males were oral, tongue, lung, and chronic myeloid leukemia while in females breast and cervical cancer. In India, the leading sites of cancers in males are the lung, esophagus, larynx, mouth, and tongue, and in females breast and cervix uteri [11]. In females and males, the data regarding top cancers in our study is comparable to National data with some regional variation seen. It is well-documented that there are heterogeneities in cancer epidemiology within India [12-15]. The differences in cancer incidence rates have been published by National Cancer Registry Program (NCRP), India. The incidence rates of cancer in males were seven times higher in the Aizawl district of Mizoram and four times in females as compared to Osmanabad and Beed in Maharashtra [15]. The crude annual incidence of all cancers in the state of Andhra Pradesh was 76.6 as reported in the year 2016 [13].

In males, 17 cancer cases were at sites with sufficient evidence of carcinogenicity related to tobacco exposure and out of them, eight (47.5%) were associated with tobacco exposure. The IARC has classified 83 tobacco and tobacco smoke components as carcinogens. Out of them, 18 were Group 1 carcinogens; 15 were classified as group 2A carcinogens; and 50 as group 2B carcinogens [16].

In our study, 6.5% (7/107) could not complete their treatment for cancer and 5.6% (6/107) cancer cases could not avail of the regular follow-up required because of financial reasons. Appropriate cancer treatment is essential to improve outcomes. But the cost of treatment can lead to extreme financial burden and distress [17].

According to GLOBOCAN estimates, the number of cancers diagnosed in India in 2012 was 1157294. But the Global Burden of Disease (GDB) India study reported 1069000 cancer cases in the same year 2012 [18]. So it is evident that there are differences in cancer cases reported by the various studies. Cancer prevalence can vary depending on the source of data and the population being studied. Factors such as age, sex, race, and lifestyle can all affect the prevalence of cancer. Additionally, different countries and regions may have different rates of cancer due to variations in healthcare access, screening practices, and risk factors. It's important to consider the specific population and data source when interpreting prevalence rates. The main limitation of the study is it covered only known cancer cases of Mangalagiri Mandal of the Guntur region, Andhra Pradesh, there are rare chances for the case to be missed if the patient does not want to reveal it.

## Conclusions

The data on cancer incidence and prevalence is essential for planning cancer control measures and monitoring progress in reducing the burden of cancer for the allocation of healthcare resources. PBCRs are important for several reasons. They provide detailed information on the incidence and distribution of cancer within a defined population, which can be used to identify patterns and trends in cancer occurrence. This information is crucial for planning and evaluating cancer control programs, as well as for monitoring progress in reducing the burden of cancer. Additionally, PBCRs provide data that can be used to evaluate the effectiveness of cancer treatments and to identify disparities in cancer outcomes among different subpopulations. Overall, PBCRs play a key role in informing public health policy and practice, and in guiding research on the causes and prevention of cancer.
